# A Novel Lightweight Anonymous Proxy Traffic Detection Method Based on Spatio-Temporal Features

**DOI:** 10.3390/s22114216

**Published:** 2022-06-01

**Authors:** Yanjie He, Wei Li

**Affiliations:** School of Computer Science and Technology, Xi’an Jiaotong University, Xi’an 710049, China; liw@xjtu.edu.cn

**Keywords:** Shadowsocks traffic detection, VPN traffic detection, spatio-temporal features, CNN

## Abstract

Anonymous proxies are used by criminals for illegal network activities due to their anonymity, such as data theft and cyber attacks. Therefore, anonymous proxy traffic detection is very essential for network security. In recent years, detection based on deep learning has become a hot research topic, since deep learning can automatically extract and select traffic features. To make (heterogeneous) network traffic adapt to the homogeneous input of typical deep learning algorithms, a major branch of existing studies convert network traffic into images for detection. However, such studies are commonly subject to the limitation of large-sized image representation of network traffic, resulting in very large storage and computational resource overhead. To address this limitation, a novel method for anonymous proxy traffic detection is proposed. The method is one of the solutions to reduce storage and computational resource overhead. Specifically, it converts the sequences of the size and inter-arrival time of the first *N* packets of a flow into images, and then categorizes the converted images using the one-dimensional convolutional neural network. Both proprietary and public datasets are used to validate the proposed method. The experimental results show that the converted images of the method are at least 90% smaller than that of existing image-based deep learning methods. With substantially smaller image sizes, the method can still achieve F1 scores up to 98.51% in Shadowsocks traffic detection and 99.8% in VPN traffic detection.

## 1. Introduction

In recent years, anonymous proxy services, e.g., Shadowsocks [1], VPN (Virtual Private Network) [2], and V2ray [3], have been used by increasingly more Internet users. On one hand, they can help users to access restricted resources by circumventing Internet censorship. On the other hand, they have become an important means for criminals to engage in illegal network activities, e.g., data theft, darknet transactions, cyber-attacks, and pornographic propagation [4]. Thus, anonymous proxy traffic detection is of great significance for network security.

Anonymous proxy traffic detection methods can be categorized into traditional machine learning methods and deep learning-based methods. Traditional machine learning methods require manually crafting and selecting features based on professional experience following a trial-and-error paradigm. This paradigm is labor-intensive and time-consuming. In recent years, the detection based on deep learning has become a hot research topic, since deep learning algorithms can automatically extract and select traffic features.

Currently, most deep learning-based methods convert the network traffic into images, for the purpose of making (heterogeneous) network traffic adapt to the homogeneous input of typical deep learning algorithms. However, these methods have a common drawback, i.e., large-sized converted images. For example, many methods (e.g., [5,6,7,8]) convert the payloads of the first few packets of a flow into an image. They connect the payloads of the first few packets of a flow into a byte stream, and then convert a byte into an integer (0 to 255). As the byte stream comprises a lot of bytes, the converted images are large, e.g., 784 bytes and 1521 bytes. The method in [9] converts the sequences of packet sizes and packet arrival time of a flow into a two-dimensional square histogram. The method extracts the size and arrival time of each packet in the flow as a record pair, and then plots the record pairs by defining the X-axis as the packet arrival time and the Y-axis as the packet size. As the MTU (Maximum Transmission Unit) is 1500 bytes, the Y-axis is set between 1 and 1500. The size of converted images is 2250 KB (1500 × 1500 pixels). Large-sized images result in very large storage and computational resource overhead.

To address the problems above, a novel method for anonymous proxy traffic detection is proposed. The method is one of the solutions to reduce storage and computational resource overhead. It converts the sequences of the size and inter-arrival time of the first *N* packets of a flow into an image, and then categorizes the converted images using the one-dimensional convolutional neural network (1D-CNN). As the method converts the two-way and one-way spatio-temporal features of a flow into an image, the method can comprehensively capture the flow differences. The method achieves comparable detection performance to the state-of-the-art methods. Meanwhile, since the method uses only a small amount of data regarding the size and inter-arrival time (rather than data including packet headers and payloads), the converted images of the method are much smaller than that of existing image-based deep learning methods. Thus, the method is very lightweight and efficient. Compared with existing image-based deep learning methods, the method can significantly reduce storage and computational resource overhead. Due to its high efficiency and low storage requirements, the method can be applied to traffic analysis tasks in large-scale networks.

To the best of our knowledge, we make the first effort towards building a lightweight image representation of encrypted network traffic, with applications to anonymous proxy traffic detection. The approach is not only lightweight, but also incorporates spatio-temporal features, thereby achieving both high efficiency and accuracy. The main contributions of this paper can be summarized below.

- A novel lightweight anonymous proxy traffic detection method is proposed. The method can convert the two-way and one-way spatio-temporal features of the flow into an image. The converted images of the method are at least 90% smaller than that of existing image-based deep learning methods, hence drastically lowering space and time computational complexity.
- Besides smaller image sizes, the proposed method achieves comparable detection performance to the state-of-the-art methods, i.e., F1 scores up to 98.51% in Shadowsocks traffic detection and 99.8% in VPN traffic detection.
- Since the proposed approach features a compact image-based representation of encrypted traffic, it could be incorporated into existing traffic analysis systems that take a mirrored copy of network traffic as input as needed. The analysis results of the systems can be further transmitted to IDS/IPS systems deployed on the network border, which then generate network management policies to allow or drop traffic.

The rest of this paper is structured as follows. Section 2 surveys the literature. Section 3 details our method, and Section 4 presents the experiments. Section 5 presents the performance comparison. We finally discuss limitations in Section 6 and conclude in Section 7.

## 2. Related Work

In this section, anonymous proxy traffic detection methods and application traffic identification methods are outlined. These methods can be divided into traditional machine learning methods and deep learning-based methods.

Traditional machine learning methods: These methods utilize handcrafted features, statistical features, and traditional machine learning algorithms to identify anonymous proxy traffic or application traffic.

Miller et al. [10] extract flow statistics including time-based statistics and other metrics, and then compile them into a dataset. They determine strongest features using Pearson’s Correlation Coefficient algorithm. They detect VPN web traffic using multi-layered perceptron neural network. Parchekani et al. [11] utilize the time-related traffic features (e.g., the forward inter-arrival time and the flow bytes per second) and the random forest algorithm to detect VPN traffic. Deng et al. [12] propose several features, e.g., the fraction of outcoming packets, the average burst length, and the time of the whole transmission. They detect Shadowsocks traffic using the Random Forest algorithm.

Zeng et al. [4] propose 12 features (e.g., the number of flow bursts and the sum of all flow burst lengths) from three aspects: the hosts’ flow behavior, the relationship between flows, and the hosts’ DNS behavior. They detect Shadowsocks traffic using the Random Forest algorithm. Cheng et al. [13] propose an active method for Shadowsocks servers detection. They collect the IP and port of the server as a dataset, and then classify servers of the Shadowsocks using machine learning algorithm XGBoost.

Shim et al. [14] use the packet order, direction, and payload size of the first *N* packets of a flow to generate unique payload size sequence (PSS) signatures for each application. They use the unique PSS signatures to identify application traffic (e.g., Skype, Outlook, and GomTV). Hajjar et al. [15] propose an identification model for network traffic application (e.g., SMTP, FTP, and MSN) identification. The identification model is based on the features (i.e., the size, the direction, and the position) of the first application-layer messages of the flow.

Deep learning-based methods: Compared with traditional machine learning methods, deep learning-based methods can automatically learn nonlinear relationships between the input and the output.

Wang et al. [16] propose a method for protocol traffic identification and anomalous protocol traffic detection. They first propose to convert network traffic into images. They connect the payload bytes of a TCP flow, and then a byte is transformed into an integer (0 to 255). They classify the converted images using Artificial Neural Network (ANN) and Stacked Auto-Encoder (SAE). Many methods improve the classification performance on the basis of the method in [16]. Tang et al. [17] propose a novel deep neural network for encrypted VPN network traffic identification. The deep neural network consists of CapsNet and Long Short-Term Memory (LSTM) network. Guo et al. [5] propose two deep learning-based models for VPN traffic detection and VPN traffic classification, i.e., convolutional auto-encoding (CAE) and convolutional neural network (CNN). Cheng et al. [6] design a lightweight model for online encrypted traffic classification. The number of parameters and training time of the model are significantly reduced.

Lan et al. [7] propose a self-attentive deep learning method for application identification and darknet traffic classification. They use a 1D-CNN and a bidirectional LSTM network to capture local spatial–temporal features from the payload content of packets. They also extract side-channel features (e.g., Number of packets/bytes per second) from payload statistics to improve the classification performance. Lotfollahi et al. [18] propose to categorize network traffic by classifying packets. They convert a packet into an image, and then classify the converted images using the stacked autoencoder (SAE) and the convolution neural network (CNN). Wang et al. [19,20] show that the performance of 1D-CNN is better than 2D-CNN in encrypted traffic classification. Hu et al. [21] propose a network for encrypted traffic classification. The network consists of CNN and LSTM.

Johnson et al. [22] use various traditional machine learning algorithms (e.g., Random Forest, Decision Tree, k-Nearest Neighbor) and Deep Neural Networks and time-based statistical features to detect tor traffic. They demonstrate that time-based features are effective for Tor traffic detection.

## 3. Method

**Figure 1:**
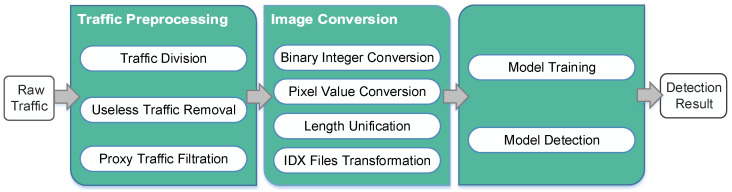
The framework of the method.

In this section, a framework is used to introduce the implementation process of the method. This framework consists of three stages, i.e., raw traffic preprocessing stage, image conversion stage, and CNN model training and detection stage. Figure 1 presents the details of the framework.

### 3.1. Feature Analysis

The first few packets of the flow are the key negotiation stage of the application. The negotiation process of the stage is based on predefined rules by the application. The key negotiation stage is different for different applications. Therefore, the size sequence of the first few packets of the flow can be used to identify application traffic [14,23].

Many anonymous proxies (e.g., Shadowsocks, V2ray, and VPN) have a similar operational mechanism. They consist of the client (Proxy-client) and the remote server (Proxy-server). The Proxy-client is generally deployed on a local machine, router, or other machines on the local network. The Proxy-server is deployed outside the firewall. The operational mechanism of anonymous proxies is as follows: the user client sends request data to the Proxy-client. The Proxy-client encrypts the request data and forwards them to the Proxy-server. The Proxy-server decrypts the request data and forwards them to the target server. The response data from the target server are returned to the original user client in the same pattern [4,12]. However, in a regular network environment, the user client sends the request data directly to the target server. The target server sends the response data directly to the user client. This comparison shows that using anonymous proxies increases the time overhead of data transmission. The packet inter-arrival time of anonymous proxy traffic is longer than that of regular traffic. Therefore, the packet inter-arrival time sequence of a flow can be used as a distinguishable feature to detect anonymous proxy traffic.

### 3.2. Image Conversion Method

The sequences of sizes and inter-arrival time of the first *N* packets of a flow are considered as a grayscale image. The size of the packet is transformed into a binary integer. Most packet inter-arrival time of regular traffic and anonymous proxy traffic is less than 1 s. Therefore, the packet inter-arrival time is converted into an integer by Equation (1), and then the integer is transformed into a binary integer. The binary integers of each feature are connected into a binary stream. The binary stream is divided into bytes (8 bits), and if there is binary data that less than a byte, 0 is added at the end of it to complement to a byte. After that, a byte is converted into an integer (0 to 255), which corresponds to a pixel value of an image.

(1) Integer=round(Time×Value),

where the *Time* is the packet inter-arrival time. The *Value* is the integer conversion value of the packet inter-arrival time (the value that transforms the packet inter-arrival time into an integer is called the integer conversion value).

CNN is used to detect anonymous proxy traffic. The images fed into the CNN must have a unified size. In this work, the size of the converted images is set based on *N* (the first *N* packets of a flow) and the MTU of the network. Specifically, to reduce data loss, the size of the converted images is set according to the special case that the size of the first *N* packets of a flow is MTU. For Shadowsocks traffic detection, the size of converted images of the method is 49 bytes (7×7 pixels). For VPN traffic detection, the size of converted images of the method is 16 bytes (4×4 pixels).

Taking the converted images of 49 bytes as an example, the construction process of the converted image is introduced. To reduce the interference between different features, the pixel value sequences of the features are unified to the same length (i.e., 7 or 14 bytes). If the sequence of pixel values is less than 7 bytes, 0 is appended to the end of it to complement to 7 bytes. If the sequence of pixel values is more than 7 bytes and less than 14 bytes, 0 is appended to the end of it to complement to 14 bytes. The pixel value sequences of all the features are connected into a sequence. If the connected sequence is less than 49 bytes, 0 is appended at the end of it to complement to 49 bytes. If the connected sequence is more than 49 bytes, it is truncated to 49 bytes. Finally, the pixel value files and the label files are transformed into IDX format files [24].

**Figure 2:**
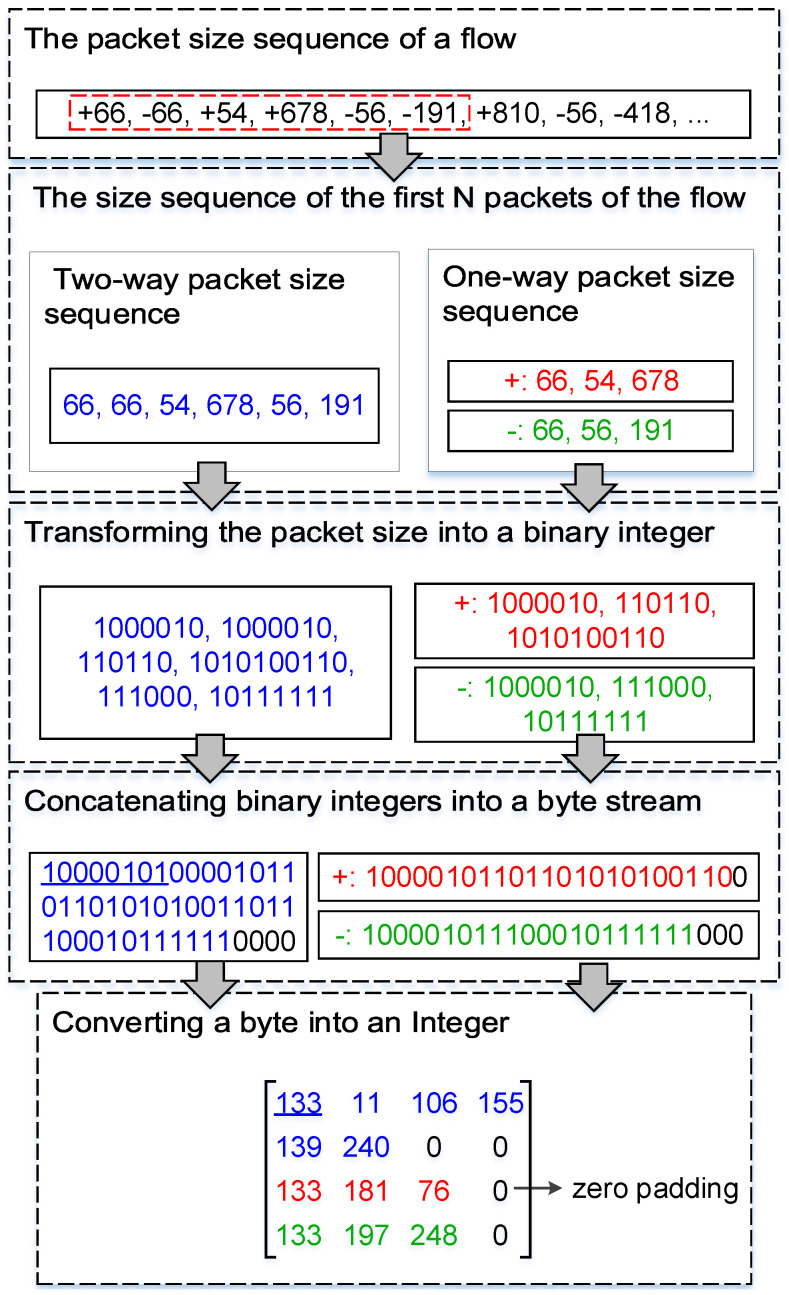
The image conversion process of the method.

The process of converting the inter-arrival time of packets into pixel values is similar to the process of converting the size of the packet into pixel values. Thus, taking the process of converting the size of the packet into pixel values of an image as an example, the image conversion process of the method is introduced. The image conversion process of the method is shown in Figure 2. In this figure, the *+* and − stand for the forward direction (client to server) and the backward direction (server to client) of the flow, respectively.

### 3.3. Cnn Model

CNNs have been widely used in the field of computer vision, such as image recognition [25,26,27] and video analysis [28,29].

The 1D-CNN model consists of 7 layers, i.e., an input layer, two convolutional layers, two pooling layers, a fully connected layer, and an output layer. The convolutional layer extracts different features of the input image by the convolution operation. The convolution operation is defined as:

(2) $$S\left(i,j\right)=\left(K*I\right)\left(i,j\right)=\sum_{m}^{}\sum_{n}^{}I\left(i-m,j-n\right)K\left(m,n\right),$$

where *I* is the input, and *K* is a kernel function. The pooling layer selects important features, which can reduce the parameters of the model. In this model, max-pooling is used. The activation function introduces the nonlinearity into the network, which improves the expressive ability of the model. In this model, the Rectified Linear Units (ReLU) activation function is used. The ReLU activation function is defined as:

(3) ReLU(x)=max(0,x),

As mentioned before, the input of the model is grayscale images of size 16 bytes (4×4 pixels) and 49 bytes (7×7 pixels). Taking the input images of 49 bytes as an example, the parameter of the model is introduced. The size of the filter of the 1-dimensional convolutional layers is 1×25. The size of the filter of the max-pooling layer is 1×3. The output of the first convolutional layer is 32 feature maps of size 1×49. The output of the first max-pooling layer is 32 feature maps of size 1×17. The output of the second convolutional layer is 64 feature maps of size 1×17. The output of the second max-pooling layer is 64 feature maps of size 1×6. The output of the fully connected layer is 1024. Moreover, the Dropout layer is used to improve the generalization ability of the model. The details of the 1D-CNN model are presented in Table 1.

## 4. Experiment

In this section, the datasets and the process of traffic preprocessing are presented. After that, metrics for evaluating the method are introduced. The parameters of the method are analyzed. The performance of the method on different versions of Shadowsocks traffic detection tasks is analyzed.

### 4.1. Datasets

Both the self-collected dataset Shadowsocks-Regular and the public dataset ISCX VPN-nonVPN [30] are used to validate the proposed approach. The self-collected dataset Shadowsocks-Regular was built by Wireshark [31]. When collecting the Shadowsocks traffic, the Shadowsocks client is set to global mode. The Shadowsocks-Regular dataset consists of regular network traffic and the network traffic via Shadowsocks. Both the regular traffic and Shadowsocks traffic consist of data of multiple popular applications.

The public ISCX VPN-nonVPN dataset consists of 14 types of network traffic: 7 types of regular network traffic (i.e., P2P, VoIP, Browsing, Streaming, Chat, File transfer, and Email) and 7 types of network traffic via VPN (i.e., VPN-P2P, VPN-VoIP, VPN-Browsing, VPN-Streaming, VPN-Chat, VPN-File transfer, and VPN-Email). Each type of traffic comprises the data of multiple popular applications. For example, the Chat traffic comprises the data of ICQ, AIM, Facebook, Hangouts, and Skype. The details of these two datasets are presented in Table 2 and Table 3.

### 4.2. Data Preprocessing

The flow is a traffic unit based on the same 5-tuple (source IP, destination IP, source port, destination port, and transport-level protocol) [7,32]. The packets in a flow are sorted by the arrival time. The raw traffic is divided into multiple flows, each of which is saved as a file. During Shadowsocks traffic collection, some regular traffic still was captured even though the Shadowsocks client was set to global mode. Thus, the regular traffic was filtered out based on the port number of the Shadowsocks server. The FIN and RST are the end marker of TCP flows. If there is no packet including the FIN or RST in the TCP flow, the end of the flow file is the termination of the flow.

Some network traffic that is useless to us was removed, such as the Domain Name System (DNS) traffic. Flows without a payload were removed. Moreover, for the ISCX VPN-nonVPN dataset, incomplete TCP flows and very small flows were removed. An incomplete TCP flow has no connection establishment phase (Three-way handshake). Flows with less than two packets with a payload are very small flows.

### 4.3. Evaluation Metrics

Four metrics are used to assess the proposed approach, i.e., Accuracy (*Acc*), Precision (*Pre*), Recall (*Rec*), and F1 Score (*F*1). These four metrics are defined as follows:

(4) $$Acc=\frac{TP+TN}{TP+FP+FN+TN}$$

(5) $$Pre=\frac{TP}{TP+FP}$$

(6) $$Rec=\frac{TP}{TP+FN}$$

(7) $$F1=\frac{2*Pre*Rec}{Pre+Rec}$$

where *FP*, *FN*, *TP*, and *TN* stand for false positives, false negatives, true positives, and true negatives, respectively.

### 4.4. Experimental Evaluation under Different Parameter Settings

The validity of the features is validated. After that, the *N* (the first *N* packets of a flow) value and the integer conversion value of the packet inter-arrival time that enable the method to achieve better performance are analyzed. The detection performance of the method using the payload size sequence of the flow is analyzed. The effect of zero-padding between different features on the performance of the method is analyzed.

#### 4.4.1. The Features

**Figure 3:**
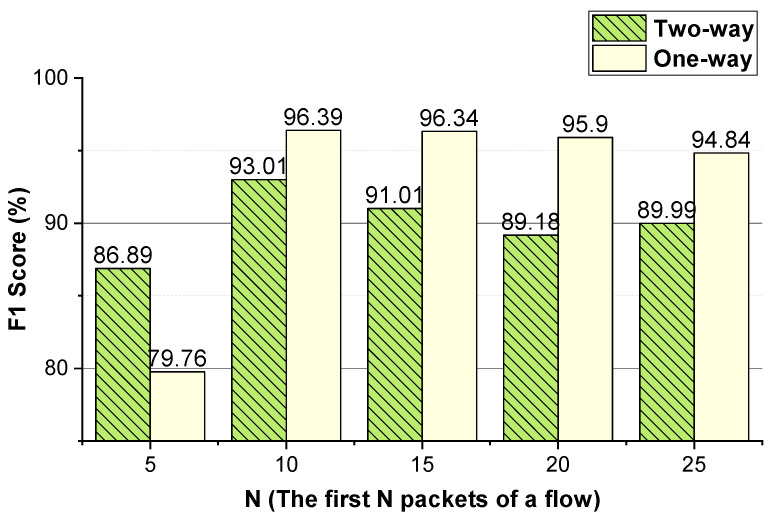
The Shadowsocks traffic detection performance of the method using the two-way and one-way packet size sequences of the flow, respectively.

**Figure 4:**
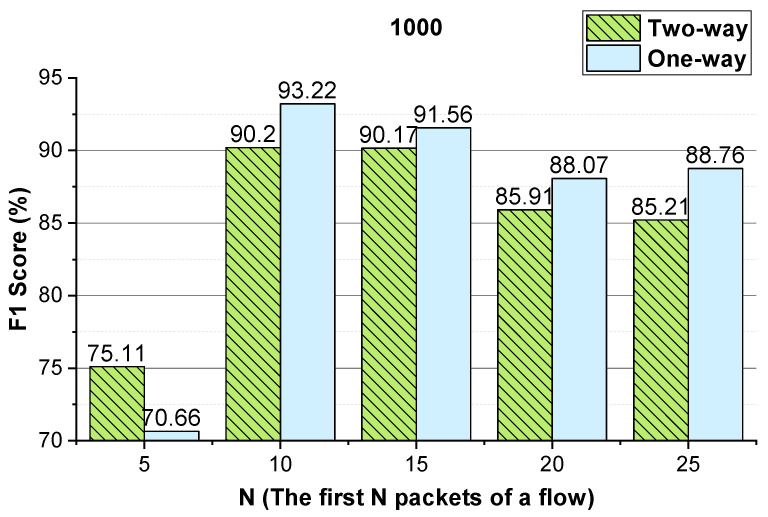
When the integer conversion value of the packet inter-arrival time is 1000, the Shadowsocks traffic detection performance of the method using the two-way and one-way packet inter-arrival time sequences of the flow, respectively.

**Figure 5:**
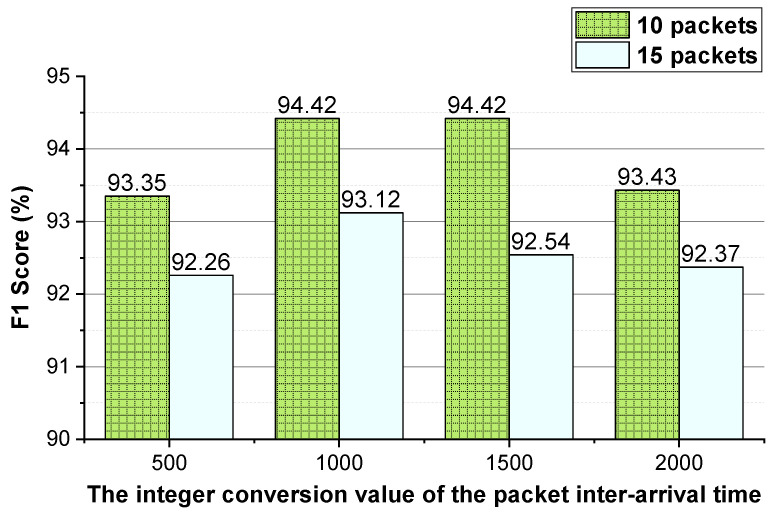
The Shadowsocks traffic detection performance of the method using different integer conversion values of packet inter-arrival time.

**Figure 6:**
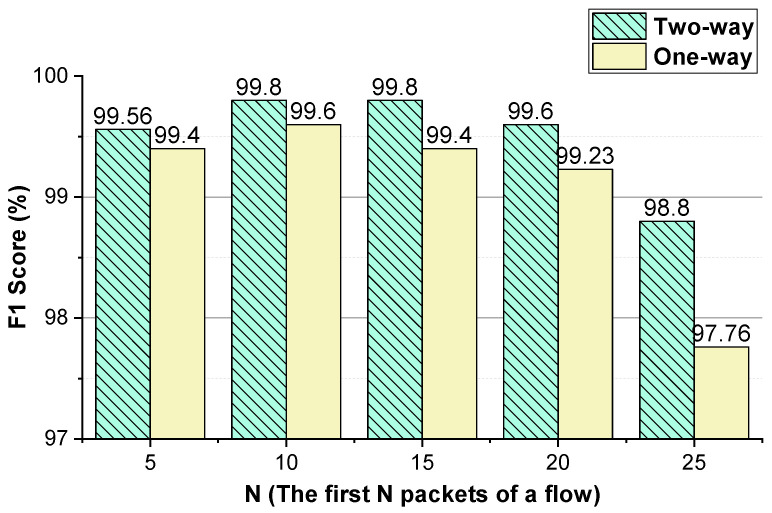
The VPN traffic detection performance of the method using the two-way and one-way packet size sequences of the flow, respectively.

**Figure 7:**
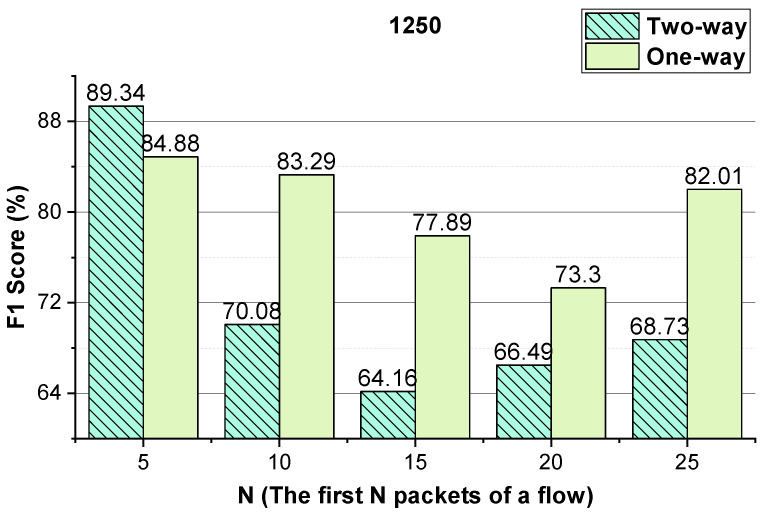
When the integer conversion value of the packet inter-arrival time is 1250, the VPN traffic detection performance of the method using the two-way and one-way packet inter-arrival time sequences of the flow, respectively.

**Figure 8:**
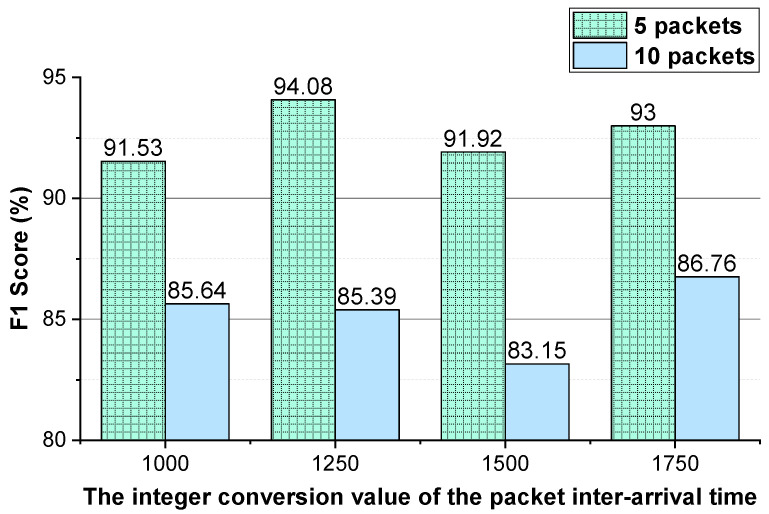
The VPN traffic detection performance of the method using different integer conversion values of packet inter-arrival time.

We evaluate the effectiveness of the features (i.e., the two-way and one-way packet size sequences of a flow and the two-way and one-way packet inter-arrival time sequences of a flow). The integer conversion value of the packet inter-arrival time and the *N* (the first *N* packets of the flow) values that enable the method to achieve better performance are analyzed. The performance of the method using temporal features of the flow on VoIP traffic and VPN-VoIP traffic classification is not good. Therefore, 12 types of network traffic of the ISCX VPN-nonVPN dataset except VoIP and VPN-VoIP traffic are used to validate the temporal features of the flow. The analysis results are presented in Figure 3, Figure 4, Figure 5, Figure 6, Figure 7 and Figure 8. In these figures, the *Two-way* stands for the two-way packet size sequence of the flow or the two-way packet inter-arrival time sequence of the flow. The *One-way* stands for the one-way packet size sequences of the flow or the one-way packet inter-arrival time sequences of the flow. The *5 packets* stands for the first 5 packets of a flow.

As shown in Figure 3, Figure 4, Figure 6 and Figure 7, the two-way and one-way packet size sequences of the flow and the two-way and one-way packet inter-arrival time sequences of the flow are effective in Shadowsocks and VPN traffic detection. Moreover, these features have optimal *N* values that enable the method to achieve the best performance. For the time features, the two-way and one-way packet inter-arrival time sequences of the flow have optimal integer conversion values that enable the method to attain the best performance.

As shown in Figure 3 and Figure 4, for Shadowsocks traffic detection, converting the two-way size sequence of the first *N* packets of a flow into an image, the approach obtains the best performance when *N* is 10. Converting the one-way size sequences of the first *N* packets of a flow into an image, when *N* is 10, the approach achieves the best performance. Converting the two-way inter-arrival time sequence of the first *N* packets of a flow into an image, the approach attains the best performance when *N* is 10. Converting the one-way inter-arrival sequences of the first *N* packets of a flow into an image, when *N* is 10, the approach attains the best performance. As shown in Figure 5, the approach achieves the best performance when the integer conversion value of the packet inter-arrival time is 1000/1500.

As shown in Figure 6 and Figure 7, for VPN traffic detection, converting the two-way size sequence of the first *N* packets of a flow into an image, the approach obtains the best performance when *N* is 10/15. Converting the one-way size sequences of the first *N* packets of a flow into an image, when *N* is 10, the approach achieves the best performance. Converting the two-way inter-arrival time sequence of the first *N* packets of a flow into an image, the approach achieves the best performance when *N* is 5. Converting the one-way inter-arrival sequences of the first *N* packets of a flow into an image, when *N* is 5, the approach attains the best performance. As shown in Figure 8, the approach achieves the best performance when the integer conversion value of the packet inter-arrival time is 1250.

Through the above analysis, for the two-way and one-way packet size sequences of the flow and the two-way and one-way packet inter-arrival time sequences of the flow, the uniform *N* value is used. For Shadowsocks traffic detection, 1000/1500 is used as the integer conversion value of the packet inter-arrival time. As the performance of the method using temporal features of the flow on VoIP traffic and VPN-VoIP traffic classification is not good, the temporal features of the flow are not used to detect VPN traffic.

#### 4.4.2. *N* (the First *N* Packets of a Flow)

**Figure 9:**
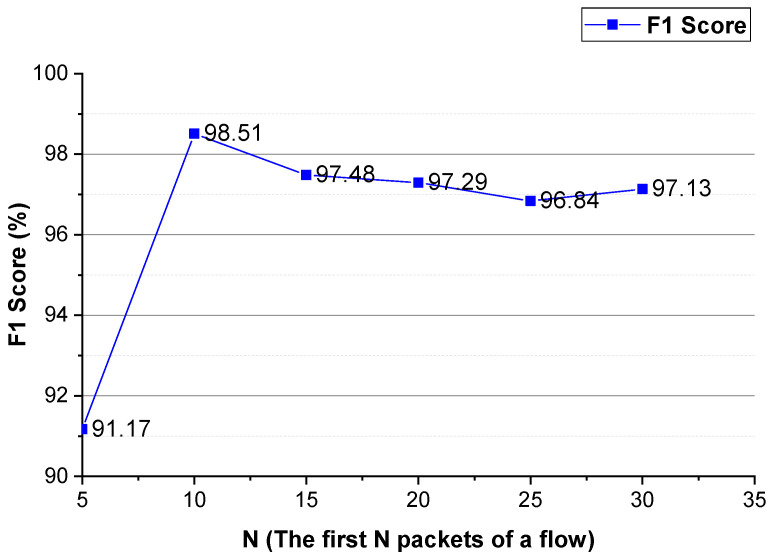
The Shadowsocks traffic detection performance of the method using different *N* values.

**Figure 10:**
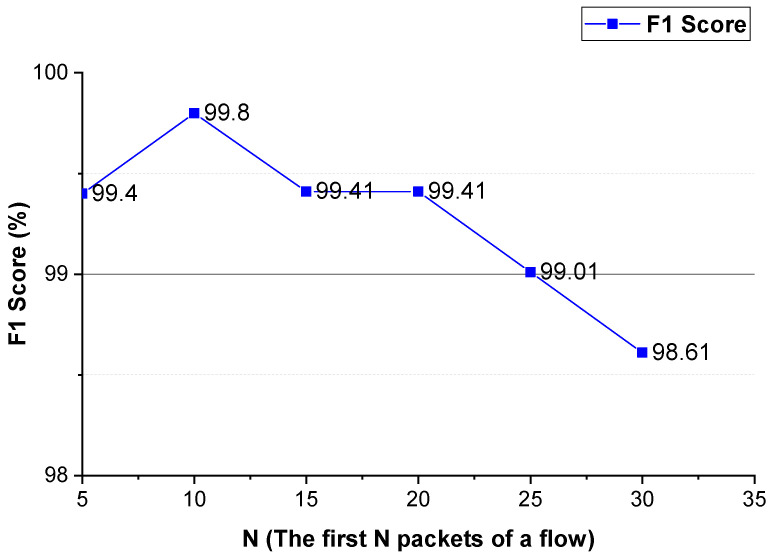
The VPN traffic detection performance of the method using different *N* values.

We evaluate the parameter *N* that enables the approach to achieve better performance. The evaluation results are shown in Figure 9 and Figure 10. As shown in these two figures, for Shadowsocks and VPN traffic detections, when the first 10 packets of the flow are used, the method achieves the best detection performance. In comparison to Figure 6 and Figure 10, for VPN traffic detection, the best performance of the method using the two-way and one-way packet size sequences of the flow is the same as that using only the two-way packet size sequence of the flow. Therefore, for VPN traffic detection, only the two-way packet size sequence of the flow is used.

These experimental results show that the first N packets (the key negotiation stage) of the flow have unique features of an anonymous proxy. The sequences of the size and inter-arrival time of the first *N* packets of the flow can be used as distinguishable features to detect anonymous proxy traffic. The two-way and one-way spatio-temporal features of the flow have different distinguishable features, they make different contributions to anonymous proxy traffic detection.

#### 4.4.3. Payload Size Sequence

We analyze the detection performance of the method using the payload size sequence of the first *N* packets of a flow. The analysis results are shown in Table 4. In this table, the *N* stands for the first *N* packets of a flow. The *Payload* stands for the payload size sequence of the flow. The *Packet* stands for the packet size sequence of the flow.

As shown in Table 4, in Shadowsocks and VPN traffic detections, the performance of the method using the packet size sequence of the flow is better than that using the payload size sequence of the flow. For Shadowsocks traffic detection, the F1 score of the method using the packet size sequence of the flow is 1.83% higher than that using the payload size sequence of the flow. For VPN traffic detection, the F1 score of the method using the packet size sequence of the flow is 19.71% higher than that using the payload size sequence of the flow. These experimental results show that the acknowledgment packet at the Key negotiation stage can also make contributions to Shadowsocks and VPN traffic detections.

#### 4.4.4. Zero Padding

We analyze the effect of zero-padding between different features on the performance of the method. Since only one feature is used in VPN traffic detection, the effect of zero-padding on VPN traffic detection is no longer analyzed. The analysis results on Shadowsocks traffic detection are shown in Table 5. In this table, the *Non-padding* means that the pixel value sequences of all features are directly connected into a sequence, without zero padding. *Zero-padding* means that the pixel value sequences of the features are unified to the same length, if the sequence is less than the uniform length, then zero-padding. *Zero-padding (More)* refers to padding more zeros between different features.

As shown in Table 5, in Shadowsocks traffic detection, the F1 score of the method using zero-padding is 1.36% higher than that without zero-padding. The F1 score of the method setting the size of converted images to 49 bytes is 0.04% higher than that setting the size of converted images to 81 bytes. It can be seen that the performance of the method using zero-padding is better than that without zero-padding. Padding more zeros between different features does not improve the detection performance.

### 4.5. Different Versions of Shadowsocks Traffic

In this section, the performance of the method on different versions of Shadowsocks (i.e., Shadowsocks and ShadowsocksR) traffic detections is analyzed. Shadowsocks traffic of different versions was collected in the same pattern. They consists of the traffic of the same applications (i.e., Youtube, Spotify, Twitter, Instagram, some posts, and some blogs). The analysis results are showed in Table 6. In this table, the *Size* represents the two-way and one-way packet size sequences of the flow. The *Time* represents the two-way and one-way packet inter-arrival time sequences of the flow. The *Value* is the integer conversion value of the packet inter-arrival time.

As shown in Table 6, the method achieves comparable performance in different versions of Shadowsocks traffic detection tasks. Moreover, the parameter settings of the method in different versions of Shadowsocks traffic detection tasks are almost the same. It can be seen that the method can be applied to different versions of Shadowsocks traffic detection tasks. The method has wide applicability and robustness.

## 5. Performance Comparison

We compare the detection performance of our approach against the state-of-the-art methods. Specifically, the proposed method is compared with the methods in [5,9] from two aspects: accuracy and the size of the converted images (Imgsize). Both methods in [5,9] are image-based deep learning methods, but the ways they convert network traffic into images are different. Both our method and the methods in [5,9] employ the public ISCX VPN-nonVPN dataset to validate the performance of the method. Therefore, we copied the experimental results from [5,9].

As shown in Table 7, for VPN traffic detection, the accuracy of our method is 0.15% higher than that of the method in [9]. The accuracy of our method is 0.02% lower than that of the method in [5]. The converted images of our method are much smaller than that of the methods in [5,9]. Thus, our method is more lightweight and efficient than the methods in [5,9]. In the same case (e.g., the same hardware devices), our method can save a lot of resource overhead, e.g., storage resource overhead, computational resource overhead, and model training and execution time overhead. The proposed method is efficient and has low storage requirements. Thus, it can work in a large-scale network environment.

## 6. Discussion

The proposed method has several limitations that may affect its applicability in certain scenarios. First, the integer conversion value of the packet inter-arrival time and the parameter *N* (the first *N* packets of a flow) are obtained empirically. Although an optimal setting can be found through experiments, empirically setting parameters is not user-friendly. Second, it is possible to convert the traffic data into a non-image form other than an image. Researchers converting network traffic into images may want to exploit this advantage of CNN, since it is well known that CNN achieves better performance in image classification, image recognition, and natural language processing. We leave these issues as open questions for future study.

## 7. Conclusions

A novel anonymous proxy traffic detection method is proposed. Benefiting from converting the two-way and one-way spatio-temporal features of a flow into an image, the method is effective and attains comparable detection performance to the state-of-the-art methods. Moreover, the method is lightweight. Compared with existing image-based deep learning methods, the size of the converted images of the method are reduced at least 90%. Thus, the method can reduce storage and computational resource overhead. Since it is based on CNN, the method can automatically extract and select features, omitting the works of manually crafting and selecting features. Experiments have shown that our approach can detect anonymous proxy traffic effectively, with the capability of detecting different versions of Shadowsocks traffic and VPN traffic. Due to its high efficiency resulting from compact image-based traffic representation, the method can be applied to traffic analysis tasks in large-scale networks.

## Figures and Tables

**Table 1:** The parameters of 1D-CNN model.

Layer	Operation	Input	Filter	Stride	Pad	Output
1	Conv+Relu	1×49	1×25	1	same	32 × (1×49)
2	maxpool	32 × (1×49)	1×3	3	same	32 × (1×17)
3	Conv+Relu	32 × (1×17)	1×25	1	same	64 × (1×17)
4	maxpool	64 × (1×17)	1×3	3	same	64 × (1×6)
5	Full connect	64 × (1×6)	–	–	none	1024
6	softmax	1024/2	–	–	none	2

**Table 2:** Shadowsocks-Regular dataset.

Categories	Applications	Number of Flows
Regular	Iqiyi, Youku, Tengxun, Bilibili, Wangyiyun, Weibo, some posts, some blogs	10,672
Shadowsocks	Youtube, Spotify, Twitter, Instagram, some posts, some blogs	10,658

**Table 3:** ISCX VPN-nonVPN dataset.

Categories	Applications	Number of Flows
VPN	ICQ, AIM, Facebook, Hangouts, Skype, VoipBuster, Email, FTPS, SFTP, Bittorrent Vimeo, Youtube, Netflix, Spotify, Firefox, Chrome	1331
nonVPN	ICQ, AIM, Facebook, Hangouts, Skype, VoipBuster, Gmail, Email, FTPS, SFTP, uTorrent, Vimeo, Youtube, Netflix, Spotify, Firefox, Chrome	2009

**Table 4:** The detection performance of the method using the payload size sequence of the flow (%).

Task	Feature	N	Precision	Recall	F1 Score
**Shadowsocks**	Payload	5	94.31	97.81	96.03
	Packet	10	97.63	98.1	97.86
**VPN**	Payload	10	86.98	74.21	80.09
	Packet	10	99.6	100	99.8

**Table 5:** The Shadowsocks traffic detection performance of the method using zero-padding and non-padding, respectively (%).

Operation	Imgsize	Precision	Recall	F1 Score
**Non-padding**	49 B	96.88	97.43	97.15
**Zero-padding**	49 B	99.32	97.71	98.51
**Zero-padding (More)**	91 B	98.75	98.19	98.47

**Table 6:** The performance of the method on different versions of Shadowsocks traffic detections (%).

Version	Feature	N	Value	Precision	Recall	F1 Score
**Shadowsocks**	Size	10	-	95.56	97.54	96.54
	Time	10	1500	93.79	93.69	93.74
	Size & Time	10 & 10	1500	98.51	97.54	98.02
**ShadowsocksR**	Size	10	-	97.63	98.1	97.86
	Time	10	1000/1500	93.71	95.14	94.42
	Size & Time	10 & 10	1000/1500	99.32	97.71	98.51

**Table 7:** Performance comparison of VPN traffic detection (%).

Methods	Imgsize	Accuracy	Precision	Recall	F1 Score
**This Paper**	**16 B**	**99.85**	**99.6**	**100**	**99.8**
Shapira et al. [9]	2250 KB	99.7	-	-	-
Guo et al. [5]	1521 B	99.87	-	-	-

## Data Availability

Not applicable.
